# Being a post-positivist is exhausting: The daunting commitment to an uncertain truth

**DOI:** 10.36834/cmej.71151

**Published:** 2020-09-23

**Authors:** Marcel F. D’Eon

**Affiliations:** 1 Medical College of Georgia, Augusta University, Augusta, USA; 2 Professor Emeritus, University of Saskatchewan, Saskatchewan, Canada

It is exhausting being a post-positivist. We believe that there is truth out there, but we can only know it imperfectly. We are driven to searching, gathering more information, questioning our biases, and trying to take other perspectives. We never actually find peace and cannot rest from our perpetual wondering and second-guessing. Dealing with relentless ambiguity and the pursuit of truth is psychologically draining. We are prisoners of an unending quest: like Ulysses, we hear the irresistible siren voices calling us to believe that we have the truth but have restrained ourselves from falling prey to their ephemeral pleasures. We are forced to eat the unholy gruel of incomplete knowledge and pervasive uncertainty. I’m weary from the struggle.

Post modernists, instead, will continue to overlook or ignore their fatal philosophical inconsistencies. They seem skeptical of all except their own beliefs in the relativism of the natural, social, and moral realms; and it is comforting. They rest peacefully in the assurance that there are no universal truths, just viewpoints, multiple perspectives that are all subjectively true, so why bother to look painstakingly when it’s not there anyway? So much easier it is to deconstruct someone else’s sandcastle than to clearly articulate what they really believe to be true. It is right, they proclaim (both recklessly and irresponsibly, it seems to me) to tear down these meta-narratives since there is no truth. Stephen Hawking’s endeavour to search for “a theory of everything” is just a fool’ game. But wait. How is it that I can so confidently criticize my post-modern colleagues? Maybe I need to learn more and conduct more research? I certainly don’t know what I don’t know but I am uncertain about the rest and I am restless because of it.

Positivists, on the other hand, are found now mostly in chemistry, physics, and math courses and rarely in sociology or linguistics. They love to exclaim that they have the answer. And indeed, they do, but only to narrow, instrumental problems that do not answer (let alone ask) the most fundamental questions of the human condition. But happy they are masterfully manipulating atoms and ailerons. A affects B affects C and then “that’s it.” Done. (I should not criticize too much since I want the plane attached to that aileron to work flawlessly, especially when I am on board.) Sweet victory and peace. But such is not the lot of the post-positivist either in academia or in the messy real world of politics and human society generally.

I have been reflecting on the spin that is coming out of the United States forcing the entire planet to tilt further on its axis and turn now from east to west so that the sun rises where we had not expected. In my dauntless and maybe self-destructive open-mindedness I was appalled to discover that even “those” perspectives and opinions, within a certain somewhat reasonable frame sort of make sense sometimes. For example, if mail-in ballots allow for fraud, then defunding the post-office will prevent fraud. I am forced to admit that some people see things differently and are not depraved monsters or morons. I liked it better when I was right and well, they were wrong. The book I read some time ago, “I’m right and you’re an idiot”^1^ did not, as hoped, fuel my self-righteous sense of superiority. For post-positivists, each statement and claim must be examined, and I must wear my critical thinking cap 24-7. It is exhausting!

What I wrote about politics and oncology and epistemology generally in the preceding paragraphs applies to the research we present here in this new issue of the CMEJ. We are adding to the literature on these topics but not rendering the final or even, perhaps, a definitive word. While the papers have been carefully read and critiqued by our dedicated peer reviewers and editors (and we all know how that shakes down^2^), we all need to humbly and diligently wonder about the truth. Will these papers make medical education better to improve patient outcomes? Therefore, read these carefully, and if you have a different or even critical perspective to contribute, please consider writing to us using the commentary or letter to the editor sections. Public discourse and argumentation draw us closer to, but never allow us to capture, the truth. The exhausting search continues.

O’Brien and team in “Exercise is Medicine Canada workshop training improves physical activity practices of physicians across Canada, independent of initial confidence level” explored whether MDs with varying degrees of confidence discussing exercise and physical activity with their patients would equally benefit from physical activity and exercise training. They found that training improved MDs’ confidence at each level of initial confidence.

“National survey of Canadian residents and program directors regarding parental leave during residency” by Willoughby and her team reviewed the perceptions and experiences of medical students as they transitioned back to residency after parental leave. They found a need for better communication between resident program directors and parents as they return to work.

In “Resident perceptions of Competency-Based Medical Education”, Mann and team interviewed residents who were not enrolled in a competency-based program to see what they perceived as advantages and disadvantages of CBME. They concluded that anticipating residents’ expectations and perceived disadvantages would allow for training programs to be better equipped for a more successful transition to CBME.

“Health research methodology education in Canadian emergency medicine residency programs: A national environmental scan” by Wang et al studied variabilities with teaching research methodology. They found through their cross-sectional survey that the Canadian EM residency programs vary considerably. They advised continued focus on the development of a standardized, national, online research methodology curriculum.

“Rapid, collaborative generation and review of COVID-19 pandemic-specific competencies for family medicine residency training” by Wooltorton and colleagues detailed the compilation of competencies needed to facilitate teaching and learning during COVID-19. Not only did they describe the necessary competencies, but they also found that the project demonstrated an innovative collaboration of medical experts as a response to the public health crisis.

“A national survey of burnout amongst Canadian Royal College of Physicians and Surgeons of Canada emergency medicine residents” by Lui and coauthors gauged levels of burnout in emergency medicine residents. Due to the high prevalence of burnout they found, they want to investigate different interventions to improve resident wellness.

“Determinants of mental health professionals’ attitudes towards recovery: A review” by Luigi and team provided a systematic review of the attitudes of health professionals towards mental health recovery. They found that the attitudes of the healthcare workers tended to be somewhat pessimistic and they gave recommendations to address the determinants related to these attitudes.

Correia and team, in “Fostering intergenerational education: an experiential learning program for medical students and older adults”, described an intergenerational learning opportunity for medical students to participate in the “Make a New Old Friend” program. While the program intended to help medical students develop the skills to care for the elderly, they found that it was mutually beneficial for both the students and the older adults.

In “’It unsticks your mind’: Using a musicians’ masterclass to introduce oncology faculty and trainees to the practice of direct observation and coaching,” Sanatani and Potvin used a cello player to demonstrate coaching and feedback. Since musicians already have a strong coaching culture, the authors found observing the cello player aided with coaching strategies in medicine.

Luc and D’Eon in “The Patient and Family Narratives seminars at the University of Saskatchewan connect health professions students with patient experiences” describe how the Patient and Family Narratives seminars bring patient perspectives to health professions students. Each session consists of a story shared by a patient, interdisciplinary small group discussions, and a question and answer period. The patient perspectives have consistently been rated very highly by the students.

In “Virtual Ice Cream Rounds: Addressing medical clerk wellness during COVID-19”, Kearney and Lukings adapted and facilitated their first virtual ice cream rounds due to COVID-19. Their results showed improved student self-reported wellness.

“Five ways to get a grip on evaluating and improving educational continuity in health professions education programs” by Lee and Ross offered five practical tips for how to evaluate and improve educational continuity. They concluded that misunderstandings and confusion could be prevented by clearly identifying and defining what is measured when carrying out program evaluation.

Dagnone and team described “Seven ways to get a grip on implementing Competency-Based Medical Education at the program level.” Through their shared experience with implementation at Queen’s University, they hope they can benefit others for future CBME change.

Liao in “The physician as person: The missing foundation in the CanMEDS roles” stressed the role of physician as person. He noted that since it was removed from CanMEDS roles, medicine has become depersonalized. Liao emphasized that “physician as person” is not an additional role, but the crux of a physician.

In “Unexpected turn of events: Completing residency in the COVID-19 era” by Danilewitz and Bahji, they considered the unique complications associated with final-year residents graduating in the midst of the COVID-19 pandemic. They reflected on the various unknowns for graduates including how to commemorate graduation, the transition from residency to independent practice, and the completion of licensing examinations.

In their article, “Fostering trust, collaboration, and a culture of continuous quality improvement: A call for transparency in medical school accreditation,” Javidan and team advocated for public reporting of accreditation results for North American medical schools. They asserted that this transparency would increase trust and engagement within the medical community.

Wagner and team suggested including a variety of perspectives to evaluate how teams function in clinical contexts in “It takes a team: Generating evidence to define and foster collective competence in health professions education.” They argued that there needs to be a variety of research methods employed to contribute to the understanding of collective competence.

In “Command economies, graduated responsibility, and Competence-Based Medical Education, Eric Prost argued that Competence-Based Medical education with too many compulsory requirements is contrary to the spirit of CBME. He urged medical educators to help residents learn how to learn rather than just checking off so many obligatory boxes

“Educating the bystander: How contributing to ward rounds as a junior doctor or medical student can be helpful in preparation for clinical responsibilities” by Sinclair and Biyani, described Sinclair’s participation in a Urology Simulation Boot Camp. At the camp, she contributed as a disruptive junior doctor. The unhelpful junior doctor she played caused lack of cohesiveness within the team. It taught her that the junior members are still valuable to the team despite being less experienced.

Abi-Rafeh and team in “COVID-19 pandemic & medical education: A medical student’s perspective” provided a medical student’s perspective on the current pandemic. They identified learning opportunities for medical students to carry into their careers ahead.

In “Wearable technology and live video conferencing: The development of an affordable virtual teaching platform to enhance clinical skills education during the COVID-19 pandemic” by Wintraub and co-authors, the authors identified device-accessory pairings compatible with live virtual conferencing technology that can be adapted for use with medical education programs during the COVID-19 pandemic. They anticipated that their study would aid in future distance clinical skills education.

“Impact of the COVID-19 pandemic on anesthesia residency education” by O’Brien and team is a work-in-progress article that seeks to answer questions about the clinical role of anesthesia residents during the COVID-19 pandemic. They hope that their study will help shape training during pandemic conditions.

“‘COVID-19 as the equalizer’: Evolving discourses of COVID-19 and implications for medical education” by van Buuren and team is a works-in-progress article that considered how the COVID-19 pandemic has highlighted or dismantled inequity for marginalized populations. They aim to use this study to encourage future medical teaching around these issues

“Residency redeployment during a pandemic: Lessons for balancing service and learning” by Claudio and team is a COVID-19 works-in-progress article about resident deployment during the pandemic. They aim to gather information about ‘lessons learned’ during COVID-19 deployment They plan to frame a larger study about the educational outcomes.

In “Teaching an educational simulation elective outside the simulation center,” Kleinheksel and Tews are responding to remote education during COVID-19. This works-in-progress article aims to determine if the simulation education elective can achieve the same objectives when done remotely; and if it could therefore be expanded to accommodate a larger number of students.

Finally, Andrew Seal, in “Modern day superheroes” presented an image about frontline COVID-19 workers. His art expresses gratitude for these workers calling them real life superheroes.

Enjoy!


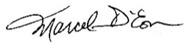
Marcel D’Eon, MEd, PhD Editor, CMEJ
